# Pseudomyxoma peritonei: An uncommon tumor

**DOI:** 10.4103/0971-5851.71657

**Published:** 2010

**Authors:** Surabhi Gupta, Garima Singh, Anurag Gupta, Hari Singh, A. K. Arya, Deepak Shrotriya, Anuj Kumar

**Affiliations:** *Department of Radiotherapy, S.N. Medical College, Agra*; 1 *Department of Pathology, S.N. Medical College, Agra*; 2 *Department of Pathology; Consultant Radiologist, S.N. Medical College, Agra*

**Keywords:** *Oral capecitabine*, *Pseudomyxoma peritonei*

## Abstract

Pseudomyxoma peritonei is a poorly understood and uncommon tumor that is known for its production of mucin in the abdominal cavity and mucinous implants, diffusely involving the peritoneal surfaces. A 60-year-old female presented to us with post-op complaints of diffuse abdominal pain and distension. On work-up, she was diagnosed as a case of Pseudomyxoma peritonei (with residual disease). She received chemotherapy in the form of oral capecitabine for residual disease. She was totally asymptomatic till the last follow-up. This case is being reported on account of its rarity and to emphasize a simple alternative treatment option as compared to the standard one.

## INTRODUCTION

Pseudomyxoma peritonei, a syndrome first described in 1842, is an enigmatic, often fatal, intra-abdominal disease characterized by disseminated gelatinous ascites and multifocal peritoneal epithelial implants, secreting copious globules of extracellular mucin.

## CASE REPORT

A 60-year-old female presented in our department in Dec 2007 with post-op complaints of diffuse abdominal pain and distension of the abdomen. On history work-up, she told the investigator that she had pre-op complaints of severe abdominal pain associated with nausea and vomiting and bleeding P/V off and on since 2 months for which she was operated on in Nov 2007 at some private hospital. Her records were checked and the surgical details revealed that she was operated in view of intestinal obstruction and on exploration of the abdomen, bilateral ovarian cysts were present with an appendicle mass and few gelatinous material on the surface of the appendix. Therefore, she underwent surgery in the form of right ovarian cyst salpingoopherectomy, left ovarian cyst excision and appendicectomy. Histopathology on gross examination showed gelatinous material within the multiloculated cyst and the appendix. The cut-surface showed dilated appedicular lumen measuring 0.9 cm and surrounding mass with cystic area containing mucinous material. Microscopic examination was suggestive of invasive well-differentiated mucinous adenocarcinoma appendix with features of Pseudomyxoma peritonei and serosal rupture, borderline (atypical proliferative) mucinous tumor bilateral ovaries and unremarkable right fallopian tube. Base of the appendix was tumor free. The ovarian tumor probably represents metastatic tumor from the appendicial primary.

The pre-op ultrasonography (USG) (26/10/07) finding revealed a 155 mm×175 mm cyst in the right ovary. The margins were well defined and multiple thick septa were seen. Small cysts were also seen in the left ovary.

**Figure 1:**
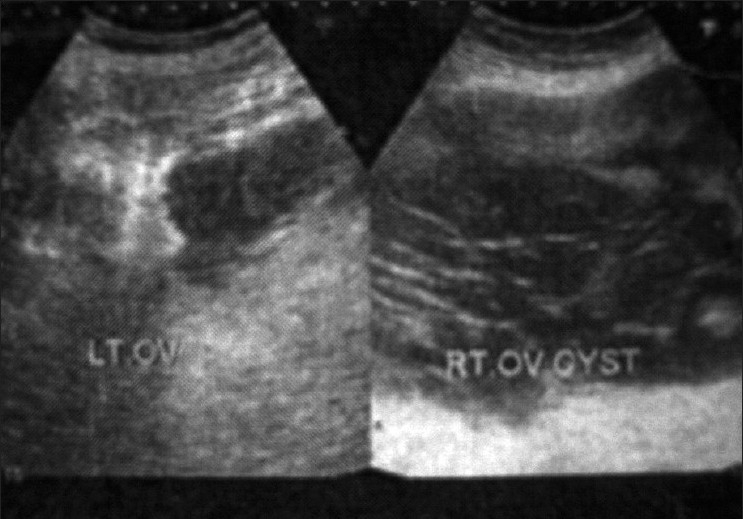
Ultrasonography of the abdomen (pre-operative) showing cystic lesion with internal septation measuring 155 mm×175 mm in the right ovary and a small cyst was also seen in the left ovary

**Figure 2:**
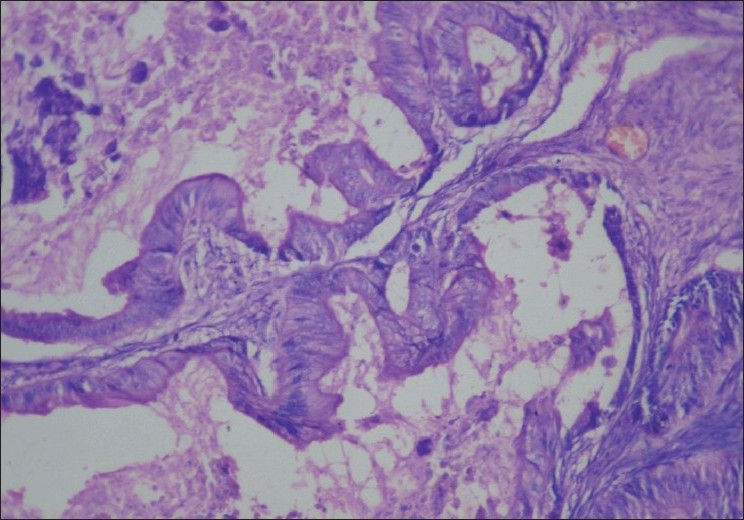
Histopathology of the ovary (operative): hematoxylin and eosin staining (×100) showing obvious nuclear atypia with complex architecture. Impression: mucino-cystadenocarcinoma of the ovary

She was properly examined. On per-abdomen examination, no organomegaly was found; the abdomen was distended, but no shifting dullness or fluid thrill was present. On per-vaginal examination, no significant finding was present No lymphadenopathy was present. She was properly investigated. Complete hemogram and liver and renal function tests were within normal limits [Figures 1–2].

Serum CEA was 5.29 U/ml, S.CA-19.9 was 2 U/ml and S.CA-125 was 2.32 U/ml. USG-guided fine needle aspiration cytology from the cyst showed gelatinous material and, microscopically, revealed mucinous adenocarcinoma.

**Figure 3:**
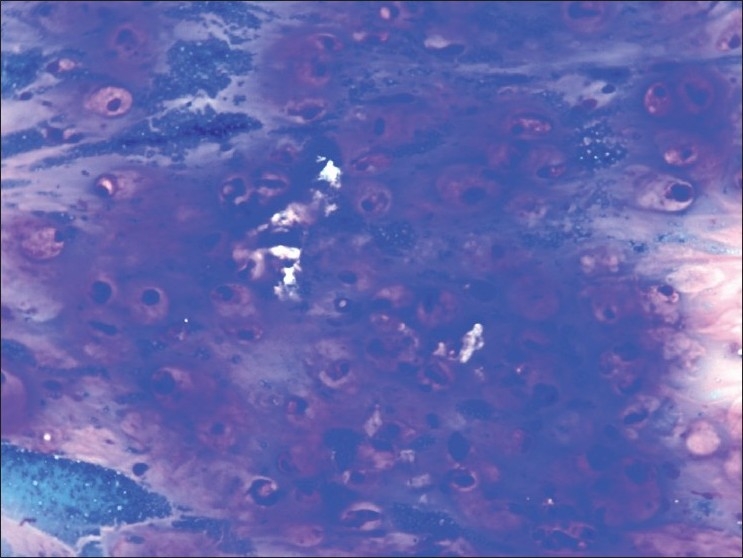
Cytology from the ovarian cyst, MGG stain (×400), showing signet ring cells and mucinosis and extramucin in the background. Impression: metastatic mucinoadenocarcinoma the ovary

Contrast-enhanced computed tomography (CECT) scan of the abdomen (17/12/07) showed a small left ovarian cyst of size 18 mm×22 mm with post-operative changes in the right lower abdomen [Figure 3].

**Figure 4:**
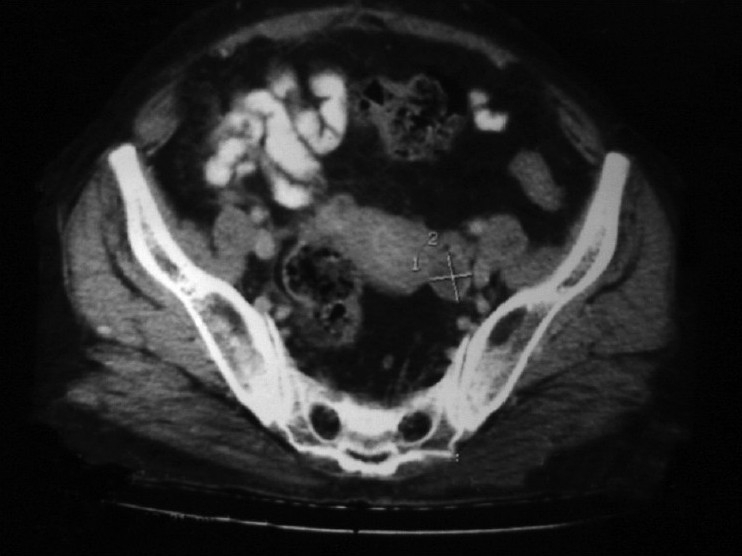
Contrast-enhanced computed tomography scan of the abdomen (pre-treatment) showing a small hypodense cystic lesion in the left adenexal region

X-ray of the chest was within the normal limit. In view of residual diseases and patient’s symptoms, she was planned for adjuvant chemotherapy with oral capecitabine 2g/day×2 weeks (q-3 weeks) in three divided doses up to six cycles. She received the 6^th^ cycle of chemotherapy on 20/04/08. On first follow-up after chemotherapy, her S.CEA-level was 3.33 U/ml and CT scan of the abdomen (17/05/08) showed that the hypodense lesion in the left adenexal region that was seen in the previous scan was not seen now [Figure 4].

**Figure 5:**
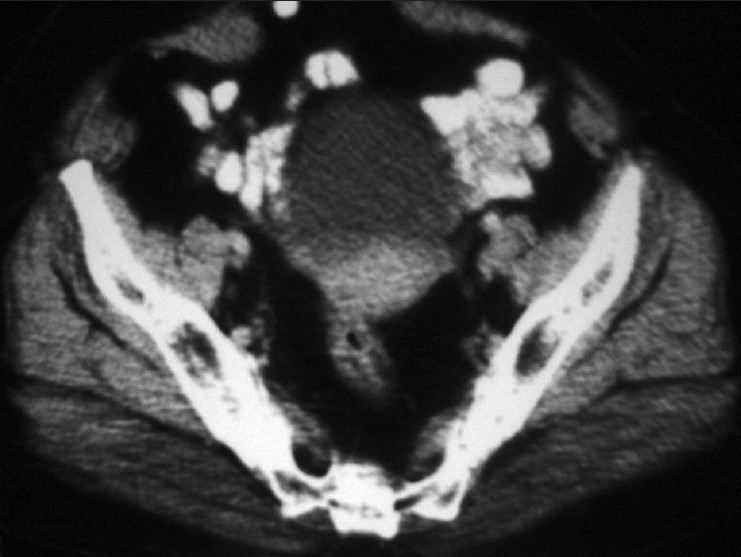
Contrast-enhanced computed tomography scan of the abdomen (post-treatment) revealed no obvious adenexal lesion in this scan, which was present on the pre-treatment scan

During chemotherapy, the patient developed incisional hernia for which herniorraphy was performed after completion of the chemotherapy cycles. The patient was kept on monthly follow-up [Figure 5].

During follow-up, she was advised to undergo monthly S.CEA. It was noticed that S.CEA was on an increasing trend from July 08 and on 26/10/09, it reached up to 5.65 U/ml. But, her CT-scan of the abdomen was normal. In view of increasing serum marker levels, the patient was advised three more cycles of capecitabine. But, due to financial problems, the patient was planned for four cycles of Inj 5-FU 500 mg I/V d1-d5 along with Inj leucovorin 50 mg I/V d1-d5 (q-4 weeks). The patient has received three cycles of chemotherapy till date and her serum CEA is 4.64 U/ml (dated 28/1/10). At present, the patient is totally asymptomatic.

## DISCUSSION

Pseudomyxoma peritonei is a disease of the MUC expressing goblet cells that are specific for mucin production. Extracellular mucin accumulates dramatically in pseudomyxoma peritonei because the number of MUC2-secreting cells dramatically increases and because this MUC2 has no place to drain[1]. The first association of gelatinous ascites with an appendiceal rupture was seen in 1901. Since then, a heterogeneous group of mucinous tumors have been identified. Mucinous appendiceal tumors were classified as disseminated peritoneal adenomucinosis, peritoneal mucinous carcinomatosis or hybrid tumor.[1]

It has been found that in most of the cases, the appendix is affected; in females, the ovaries are usually also involved. Focal proliferation appears to be a prognostic factor.[2]

Measurement of the tumor markers S.CEA and CA19.9 is useful in evaluating therapy in patients with Pseudomyxoma peritonei treated with cytoreductive surgery and heated intraperitoneal chemotherapy (HIPEC). These markers also have a prognostic value for predicting recurrent disease.[3]

The prognosis of Pseudomyxoma peritonei has always been extremely guarded. Sugarbaker published data showing that peritoneal carcinomatosis, regardless of primary tumor type, has always been a lethal condition. Recently, special treatments using cytoreductive surgery with peritonectomy procedures combined with peri-operative intraperitoneal chemotherapy have resulted in long-term survival. Pseudomyxoma peritonei may be especially appropriate for these aggressive local regional treatments.[4]

Witkamp, while treating 46 patients of Pseudomyxoma peritonei observed that Pseudomyxoma peritonei remains a fatal disease. However, extensive surgical cytoreduction combined with intra-operative HIPEC has recently emerged as a new treatment modality, which might improve survival.[5]

Deraco treated 35 patients of Pseudomyxoma peritonei. In all cases, cytoreductive surgery was performed with peritonectomy procedure. The closed abdomen technique was employed for intraperitoneal hyperthermic perfusion (IPHP) with the use of cisplatin (25 mg/m^2^/L) plus mitomycin-C (3.3 mg/m^2^/L) for 60 min under hyperthermic conditions (42.5°C), and he concluded that cytoreductive surgery associated with IPHP permitted complete tumor removal with an acceptable morbidity and mortality for patients with Pseudomyxoma peritonei. This study confirms the efficacy of the combined treatment in terms of long-term survival and local disease control.[6]

In the literature, only a limited number of patients have received adjuvant systemic chemotherapy.[7] There is only one published report showing the effectiveness of oral capecitabine in Pseudomyxoma peritonei patients, which showed the utility of oral capecitabine in recurrence after cytoreductive surgery followed by intra-operative intraperitoneal hyperthermic mitomycin-C (IPHC). Capecitabine had significantly reduced the patient’s tumor marker level and controlled the recurrent disease.[8] Capecitabine is an orally administered precursor of 5’deoxy-5-fluorouridine. It is absorbed as the intact molecule through the intestinal mucosa and undergoes activation to fluorouracil in three metabolic steps.[9] Our patient had residual disease after cytoreductive surgery at presentation, for which she was offered oral capecitabine. After six cycles, the patient was totally asymptomatic but due to rising tumor marker levels, she was further given I/V chemotherapy in the form of Inj leucovorin and 5-FU. In our case, capecitabine showed a good result with acceptable toxicity. The patient was asymptomatic till the last follow-up.

## CONCLUSION

Oral capecitabine can be considered as an alternative chemotherapy in an adjuvant setting with acceptable toxicity thus minimizing the cost burden as well as the treatment complexity, and 5-FU and leucovorin can be kept as maintenance therapy for Pseudomyxoma peritonei cases.
